# Proton Transfer and Nitro Rotation Tuned Photoisomerization of Artificial Base Pair-ZP

**DOI:** 10.3389/fchem.2020.605117

**Published:** 2020-11-30

**Authors:** Xixi Cui, Yu Zhao, Zhibing Li, Qingtian Meng, Changzhe Zhang

**Affiliations:** Shandong Province Key Laboratory of Medical Physics and Image Processing Technology, School of Physics and Electronics, Shandong Normal University, Jinan, China

**Keywords:** proton transfer, potential energy surface, photoisomerization, artificial bases, *ab initio*

## Abstract

Recently, the successful incorporation of artificial base pairs in genetics has made a significant progress in synthetic biology. The present work reports the proton transfer and photoisomerization of unnatural base pair ZP, which is synthesized from the pyrimidine analog 6-amino-5-nitro-3-(1-β-D-2′-deoxyribo-furanosyl)-2 (1H)-pyridone (Z) and paired with its Watson-Crick complement, the purine analog 2-amino-8-(1′-β-D-2′- deoxyribofuranosyl)-imidazo[1,2-a]-1,3,5-triazin-4(8H)-one (P). To explain the mechanism of proton transfer process, we constructed the relaxed potential energy surfaces (PESs) linking the different tautomers in both gas phase and solution. Our results show that the double proton transfer in the gas phase occurs in a concerted way both in S_0_ and S_1_ states, while the stepwise mechanism becomes more favorable in solution. The solvent effect can promote the single proton transfer, which undergoes a lower energy barrier in S_1_ state due to the strengthened hydrogen bond. In contrast to the excited state ultrafast deactivation process of the natural bases, there is no conical intersection between S_0_ and S_1_ states along the proton transfer coordinate to activate the decay mechanism in ZP. Of particular relevance to the photophysical properties, charge-transfer character is obviously related to the nitro rotation in S_1_ state. We characterized the molecular vibration effect on the electronic properties, which reveals the electronic excitation can be tuned by the rotation-induced structural distortion accompanied with the electron localization on nitro group.

## Introduction

In the field of biochemistry, the four natural nucleotide letters [guanine (G), cytosine (C), adenine (A), and thymine (T)] can encode virtually all genetic information. Their selective pairings to form two base pairs (i.e., AT and CG) through complementary hydrogen-bond formation underlie the storage and retrieval of all biological information. Expanding the two-base-pair genetic alphabet has been a quest in the design of artificial life since the DNA structures were discovered, followed by the mechanism of genetic material transfer being understood during cell replication (Watson and Crick, 1953a,b). In the past few years, Romesberg et al. reported a class of unnatural base pairs formed between nucleotides containing hydrophobic nucleobases, successfully replicating artificial base pairs *in vivo* (McMinn et al., 1999; Tae et al., 2001; Malyshev et al., 2014). Moreover, they have optimized different components of semisynthetic organisms by using genetic and chemical approaches, eventually making them grow robustly and be capable of storing the increased information unrestrictedly in practice (Zhang et al., 2017). In 2019, Benner and collaborators doubled the number of life's building blocks, creating a synthetic, eight-letter language (G, C, A, T, and Z, P, B, S) (Hoshika et al., 2019), which forms the base pairs through hydrogen bonding interactions and seems to store and transcribe information (Geyer et al., 2003; Yang et al., 2006; Kim et al., 2014; Zhang et al., 2015; Biondi and Benner, 2018). Previous studies demonstrated that the artificial base pairs can not only mimic the natural base pairs in terms of both structure and stability (Chawla et al., 2016), but also present novel characters in contrast to the natural bases. For example, it has been revealed that DNA strands containing artificial ZP base pairs could better combine with breast cancer cells by exponential enrichment experiment and thus can be transformed into “cancer cell hunters” (Sefah et al., 2014).

Current advances in the synthesis of artificial bases require further insight into the stability and photochemistry properties of the additional genetic code. As is known, the proton and electron transfer play an important role in regulating the properties of the system, and therefore attract widespread attention both in experimental and theoretical studies (Florián and Leszczyński, 1996; Guallar et al., 1999; Sauri et al., 2013; Bull and Thompson, 2018; Zhao et al., 2018a,b; Gonzalez-Garcia et al., 2019; Liu et al., 2019; Cheng et al., 2020; Liu S. S. et al., 2020). Especially, the proton transfer between two pairing bases acts as a key part in many biological and chemical phenomena and processes, like genetic mutation, radiation-induced DNA damage and dynamics of charge transfer in DNA. For example, Jośe Ortega et al. studied the double proton transfer of GC base pair in B-DNA and illustrated the influence of DNA biological environment on the stability of the genetic code (Soler-Polo et al., 2019). In addition, another work reports the DNA damage due to hydrogen-bonded proton transfer in the protonated GC base pairs (Lin et al., 2011). Recently, one steered molecular dynamic simulation presents that the tautomerization of the T^*^-A^*^ mispair via double proton transfer is an effective pathway of the T-A to C-G transition (Tolosa et al., 2020).

**Scheme 1:**
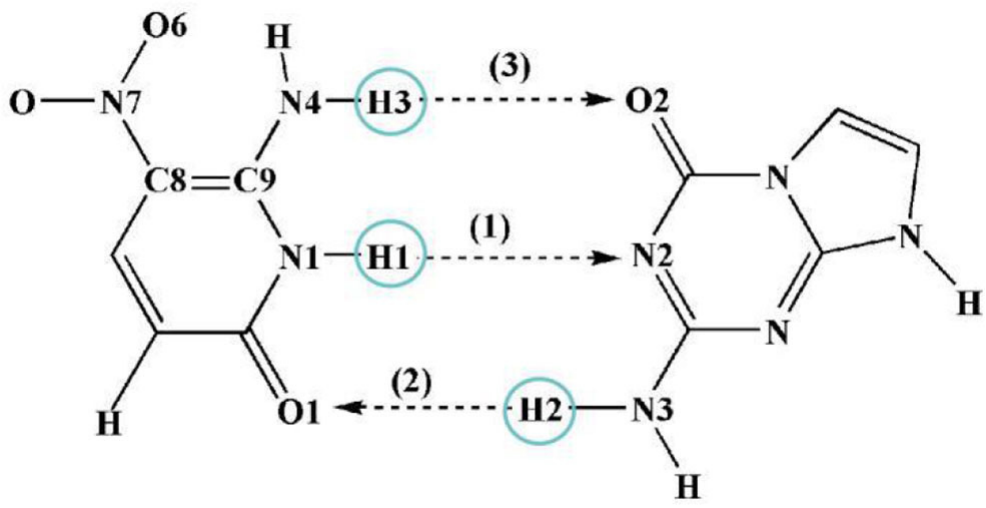
View of ZP with corresponding atom labels involved in three possible hydrogen-bonds.

Although great efforts have been made to study the proton transfer reaction of natural base pairs, to the best of our knowledge, the tautomerism of artificial bases are still poorly understood. On the basis of the mentioned above, we presented a detailed theoretical study on the proton transfer process and photoisomerization of the artificial bases ZP, paired by the pyrimidine analog 6-amino-5-nitro-3-(1-β-D-2'-deoxyribofuranosyl)-2(1H)-pyridone (Z) and its Watson-Crick complement, the purine analog 2-amino-8-(1'-β-D-2'-deoxyribofuranosyl)-imidazo[1,2-a]-1,3,5-triazin-4(8H)-one (P) (Yang et al., 2007, 2011; Chen et al., 2011), as shown in Scheme 1. It is found that the double proton transfer in the gas phase is a concerted mechanism both in ground (S_0_) and the first excited (S_1_) states, while the process in solution turns to be stepwise along the S_0_-PES and only single proton transfer is available in the S_1_ state. The solvent effect is not only conducive to the single proton transfer but also beneficial to the stabilization of related ZP products in both S_0_ and S_1_ states. Compared with the case in S_0_ state, the proton transfer undergoes a low energy barrier reaction pathway in S_1_ state due to the hydrogen bond enhancement.

Previous studies revealed that the existence of conical intersection of natural bases leads to highly efficient radiationless deactivation pathways, which returns the molecules to their ground states before chemical reactions in the excited states can lead to profound damage, and thus endow additional photostability of the natural base pairs (Sobolewski and Domcke, 2004; Sobolewski et al., 2005; Groenhof et al., 2007; Schwalb et al., 2009). However, there is no conical intersection between S_0_ and S_1_ states along the proton transfer coordinate to activate this decay mechanism in ZP. Clearly, the artificial base pair ZP has a longer lifetime in the S_1_ state and possesses weaker photostability than that of natural base pair GC.

Furthermore, our results show that the optimized configuration of ZP in S_1_ state is a non-planar structure induced by the nitro rotation accompanied with electron localization on nitro group, which indicates the lowest excited singlet state of ZP possesses charge-transfer character. It is well-known that the molecular distortion induced by the vibration is an inherent property of a molecule, which can also be activated by external energy pulse and lead to the changes of the geometric as well as the electronic properties (Huynh and Meyer, 2007; Sánchez-Carrera et al., 2010; Zimmerman et al., 2013; Feng et al., 2016; Zhang et al., 2016). Accordingly, it is worth illustrating the influence of nitro rotation on the absorption spectra and electron distribution of the system. It can be seen that the absorption maximum of ZP presents continuous red-shift associated with the rotation-induced electron localization on nitro group, and thus broadens the absorption spectrum compared with the case of natural base pairs. Our work demonstrates the proton transfer mechanism and the photophysical behaviors of artificial bases to evaluate the stability of artificial bases as the genetic code. The corresponding results can also serve as an impetus for the design of target drug based on unnatural bases.

## Computational Details

Here, the structures of ZP base pair in S_0_ and S_1_ states were fully optimized by using the DFT and TD-DFT methods at the B3LYP-D3(BJ)/ 6-311++G(d,p) level (Lee et al., 1988; Becke, 1993; Theilacker et al., 2011), which have been successful applied in other similar systems (Mazurkiewicz et al., 2006; Jiao et al., 2009). Corresponding calculations were also confirmed by utilizing the wB97-XD (Chai and Head-Gordon, 2008) and M06-2X (Zhao and Truhlar, 2008) functionals and the wave function methods (DLPNO-STEOM-CCSD and CASSCF) (Andersson et al., 1990, 1992; Hald et al., 2000; Schreiber et al., 2008; Triandafillou and Matsika, 2013). In this work, the bulk solvent (water) has been modeled with the integral equation formalism polarizable continuum model (IEFPCM) (Mennucci et al., 1997; Tomasi et al., 2005). All molecular configurations were verified to be local minima with no imaginary frequency and no constrain of bond lengths and angles were adopted except for additional remarks. To illustrate the proton transfer mechanism, the relaxed PESs linking the different tautomers in both gas and solution were constructed by scanning the bond length of N1-H1 and H2-N3 with a step of 0.05 Å. Subsequently, to adequately investigate the intermolecular hydrogen bonding interactions of ZP system, the infrared (IR) spectra, reduced density gradient (RDG), topological properties and electrostatic potential (ESP) were calculated based on optimized structures and visualized by Multiwfn program (Lu and Chen, 2012). Moreover, the variation tendencies of the electron distribution, orbital energies and absorption spectrum with the nitro rotation were also presented by utilizing the single-point calculation based on rotation-distorted configurations. The electronic excitation types were confirmed from the hole-electron distributions drawn by the Multiwfn program (Liu Z. Y. et al., 2020). The C_hole_-C_ele_ diagram is employed to smooth out the complex isosurfaces of hole and electron distribution. All calculations were carried out by the Gaussian 16 suite of programs (Frisch et al., 2016), except that the DLPNO-STEOM-CCSD method was completed by the ORCA package (Neese, 2012, 2017).

## Results and Discussion

Three possible hydrogen-bonded proton-transfer pathways of ZP base pair are considered as shown in Scheme 1. H1 proton transfer from the N1 site of Z base to the N2 site of P base is defined as the SPT1. The H2 proton transfer from the N3 site of P base to the O1 site of Z base is viewed as SPT2. Meanwhile, the H3 proton transfer from the N4 site of Z base to the O2 site of P base is regarded as SPT3. Our calculated results reveal that the SPT1 is the most common route followed by SPT2, however, SPT3 is unavailable in both S_0_ and S_1_ states.

### Proton Transfer in the Gas Phase

**Figure 1:**
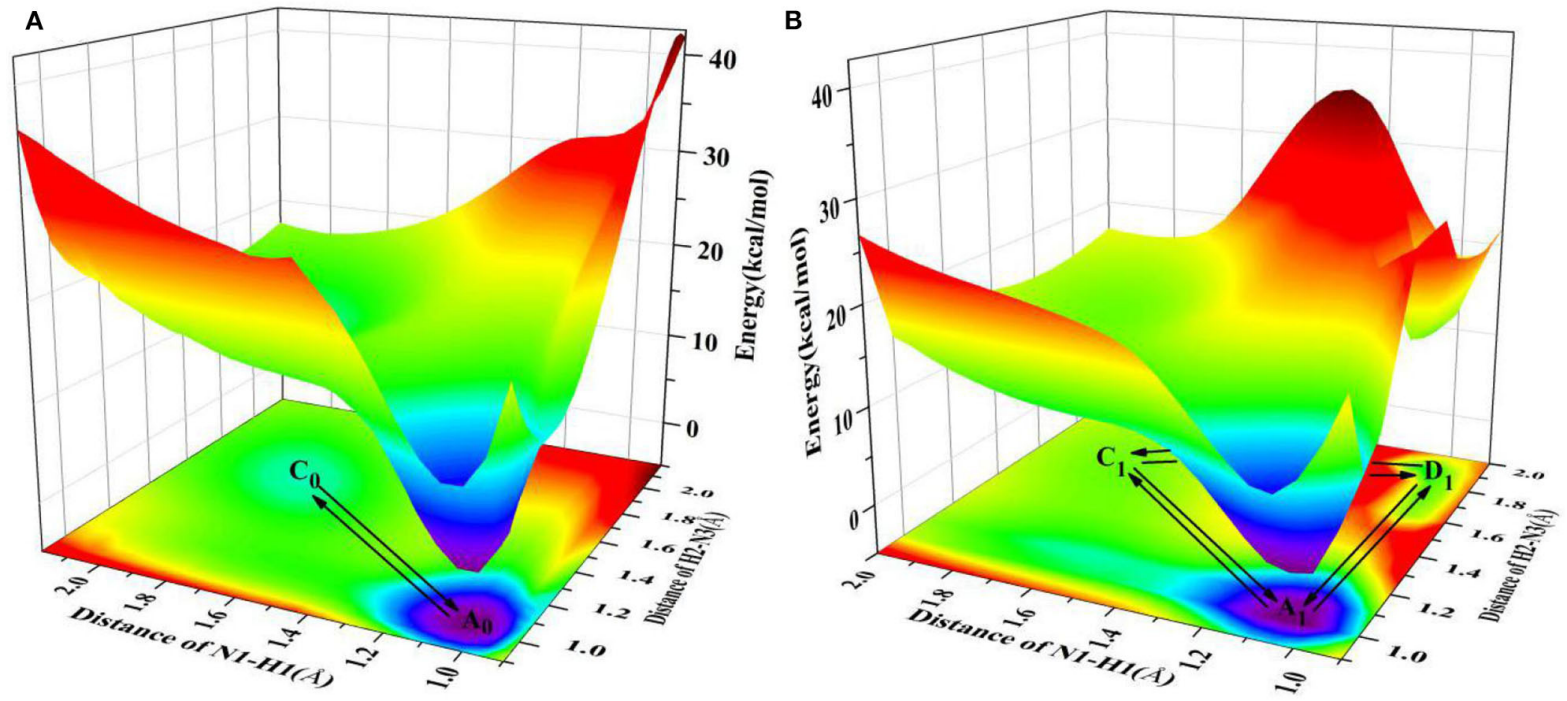
PESs on both S_0_ and S_1_ states of ZP as the functions of N1-H1 and H2-N3 length in the gas phase. **(A)** S_0_ state PES; **(B)** S_1_ state PES.

Since the main purpose of the present work is to investigate the proton transfer reaction and photophysical properties of ZP in solution, it is helpful to first know relevant basic information about ZP in the gas phase. To this end, the gaseous mechanism of proton transfer is firstly demonstrated in detail by constructing the PESs of the S_0_ and S_1_ states as functions of N1-H1 and H2-N3 bonds shown in Figure 1. The structures of normal ZP (A_0_, A_1_), dual-proton transfer ZP-DPT (C_0_, C_1_), and single-proton transfer ZP-SPT2 (D_1_) are obtained (Supplementary Figure 1 and Supplementary Table 1), with a subsequent vibrational frequency analysis to ensure the validity of these structures. As shown in Figure 1, the energies of structure A_0_ and A_1_ are the minima in the S_0_ and S_1_ states, respectively, which indicates the canonical Watson-Crick configuration is the most stable whether in S_0_ or S_1_ state. Subsequently, it is realized that the double-proton-transfer process can take place in both S_0_ and S_1_ states. As shown in Table 1, the concerted mechanism is recognized in S_0_ state with a reaction barrier of 16.8 kcal/mol, and the reverse barrier is 4.6 kcal/mol. However, there are two possible paths in the S_1_ state: the stepwise reaction undergoes $\text{ZP}\underset{9.3\;\text{kcal}/\text{mol}}{\overset{23.4\;\text{kcal}/\text{mol}}{⇆}}\text{ZP-SPT}2\underset{14.9\;\text{kcal}/\text{mol}}{\overset{13.6\;\text{kcal}/\text{mol}}{⇆}}\text{ZP-DPT}$ and the concerted path involves the transfer of $\text{ZP}\underset{2.8\;\text{kcal}/\text{mol}}{\overset{15.6\;\text{kcal}/\text{mol}}{⇆}}\text{ZP-DPT}$. Above discussion reveals that the double-proton-transfer in both S_0_ and S_1_ states is a concerted process, however, the product ZP-DPT is unstable due to the low reverse reaction barrier. On the other hand, the single-proton-transfer can occur in S_1_ state and the 9.3 kcal/mol reverse barrier allows the product ZP-SPT2 sufficiently long-lived.

**Table 1:** The potential barrier of proton transfer process (ΔE) and reverse reaction (ΔE_re_) in the gas phase and solution.

	**Structure**	**Reaction mechanism**	**ΔE(ΔE**_**re**_**) (kcal/mol)**		
			**SPT1^a^**	**SPT2^b^**	**DPT^c^**
Gas phase	WC-ZP-S_0_	Concerted	/	/	16.8 (4.6)
	WC-ZP-S_1_	Concerted	/	/	15.6 (2.8)
		Stepwise	/	23.4 (9.3)	13.6 (14.9)
Aqueous phase	WC-ZP-S_0_	Concerted	/	/	18.6 (5.9)
		Stepwise	8.9 (1.7)	/	5.8 (0.2)
	WC-ZP-S_1_	Stepwise1	5.9 (3.2)	/	/
		Stepwise2	/	25.2 (0.17)	/

a *H1 proton transfer from the N1 site of Z base to the N2 site of P base*.

b *H2 proton transfer from the N3 site of P base to the O1 site of Z base*.

c *Double-proton transfer with H1 and H2*.

### Proton Transfer in Solution

**Figure 2:**
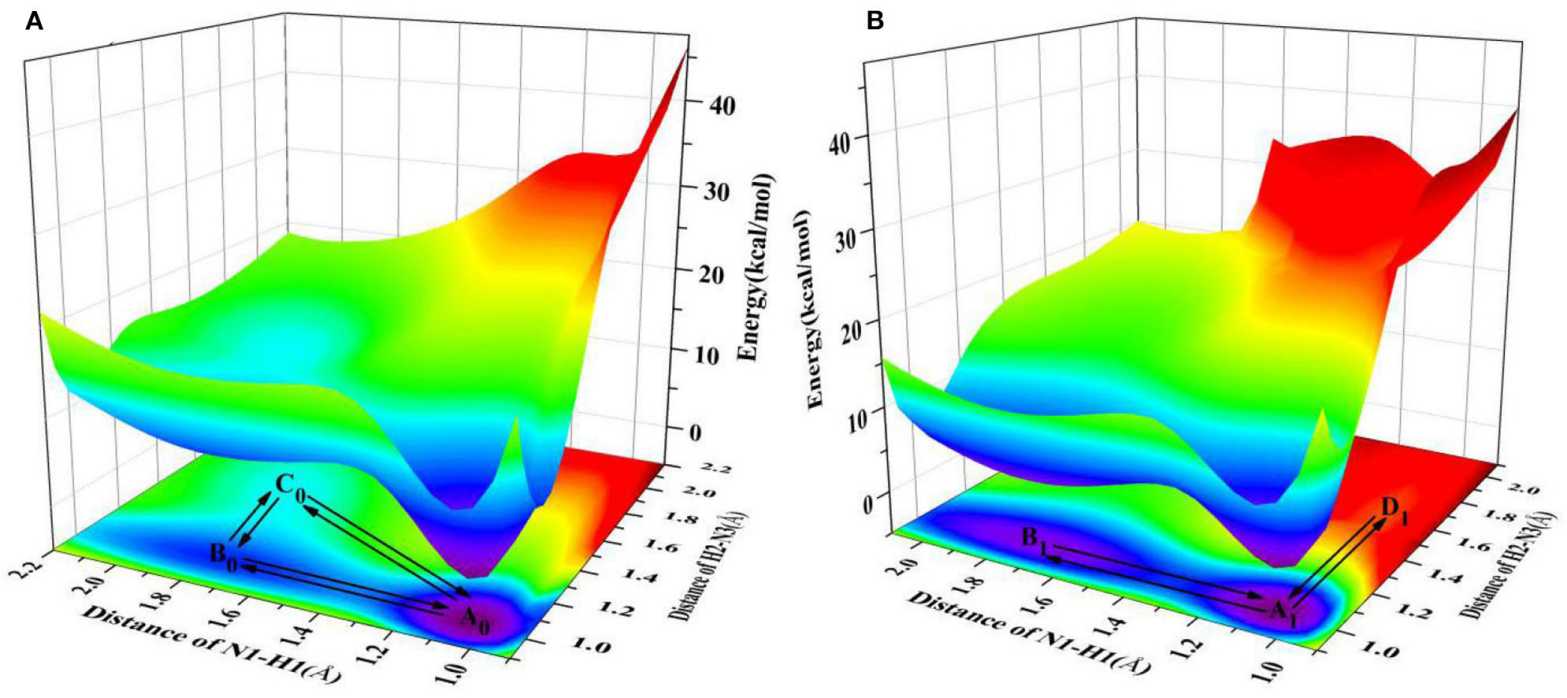
PESs on both S_0_ and S_1_ states of ZP as the functions of N1-H1 and H2-N3 length in the aqueous phase. **(A)** S_0_ state PES; **(B)** S_1_ state PES.

It is well-known that the biological micro-surrounding or aqueous solution plays an important role in the proton transfer process, which has been investigated both experimentally and theoretically (Adhikary et al., 2009; Kumar and Sevilla, 2009; Ceron-Carrasco et al., 2011). Therefore, it is necessary to consider the solvent effect in the extended calculations to explore the mechanism of proton transfer for the ZP base pairs in biological environment. Here, the SPT3 is unavailable in both S_0_ and S_1_ states (Supplementary Figure 3), and thus we constructed the 2-dimension relaxed PESs with respect to the N-H bond length of ZP in S_0_ and S_1_ states. As shown in Figure 2, there are six local minimum points in the PESs, namely A_0_, B_0_, C_0_ (S_0_), and A_1_, B_1_, D_1_ (S_1_), and the related geometric configurations are displayed in Supplementary Figure 2 and Supplementary Table 2. Similar to the case in gas phase, the configurations A_0_ and A_1_ are the most stable in S_0_ and S_1_ respectively, revealed by the corresponding energies of reactants and products listed in Supplementary Table 3. As shown in Figure 2A, there are two paths for the double proton transfer in the S_0_ state: $\text{ZP}\underset{5.9\;\text{kcal}/\text{mol}}{\overset{18.6\;\text{kcal}/\text{mol}}{⇆}}\text{ZP-DPT}$ and $\text{ZP}\underset{1.7\;\text{kcal}/\text{mol}}{\overset{8.9\;\text{kcal}/\text{mol}}{⇆}}\text{ZP-SPT}1\underset{0.2\;\text{kcal}/\text{mol}}{\overset{5.8\;\text{kcal}/\text{mol}}{⇆}}\text{ZP-DPT}$. Obviously, the stepwise process is more likely to happen than the concerted mechanism in S_0_ state. It can also be seen that the product ZP-DPT is unstable due to the shallow potential energy well. In addition, there are two single proton transfer pathways in S_1_ state (Figure 2B), which are $\text{ZP}\underset{3.2\;\text{kcal}/\text{mol}}{\overset{5.9\;\text{kcal}/\text{mol}}{⇆}}\text{ZP-SPT}1$ and $\text{ZP}\underset{\text{0}\text{.17 kcal/mol}}{\overset{\text{25}\text{.2 kcal/mol}}{⇆}}\text{ZP - SPT2}$. For the latter one, it takes place through a high energy barrier of 25.2 kcal/mol, and the whole process is endothermic by 25.0 kcal/mol. It is important to note that the barrier for the reverse process is only 0.17 kcal/mol, which indicates the energy well of the product is quite unlikely to support any bound states, so we speculate that the ZP→ZP-SPT2 process will not occur in the S_1_ state. On the other hand, the energy of the product ZP-SPT1 is just 2.7 kcal/mol higher than that of the reactant (Table 1 and Supplementary Table 3), and the obvious potential well in Figure 2A shows that the ZP-SPT1 is relatively stable in the S_1_ state. Parenthetically, the H1 atom of the N-H···N fragment is firstly transferred in both S_0_ and S_1_ states. Also, the reverse barrier of the ZP→ZP-SPT1 process is a non-negligible obstacle, so once the ZP-SPT1 configuration is formed, it will probably survive for a relatively long period. It should be noticed that the potential barrier in S_1_ state is 3.0 kcal/mol lower than that of S_0_ state, which indicates an easier transfer of proton H1 in S_1_ state. Above discussions are reproduced with considering the explicit solvent molecules (5 H_2_O molecules) by using the DFT and TD-DFT methods at the B3LYP-D3(BJ)/ 6-311++G(d,p) level as shown in Supplementary Figure 4 and Supplementary Table 4.

**Figure 3:**
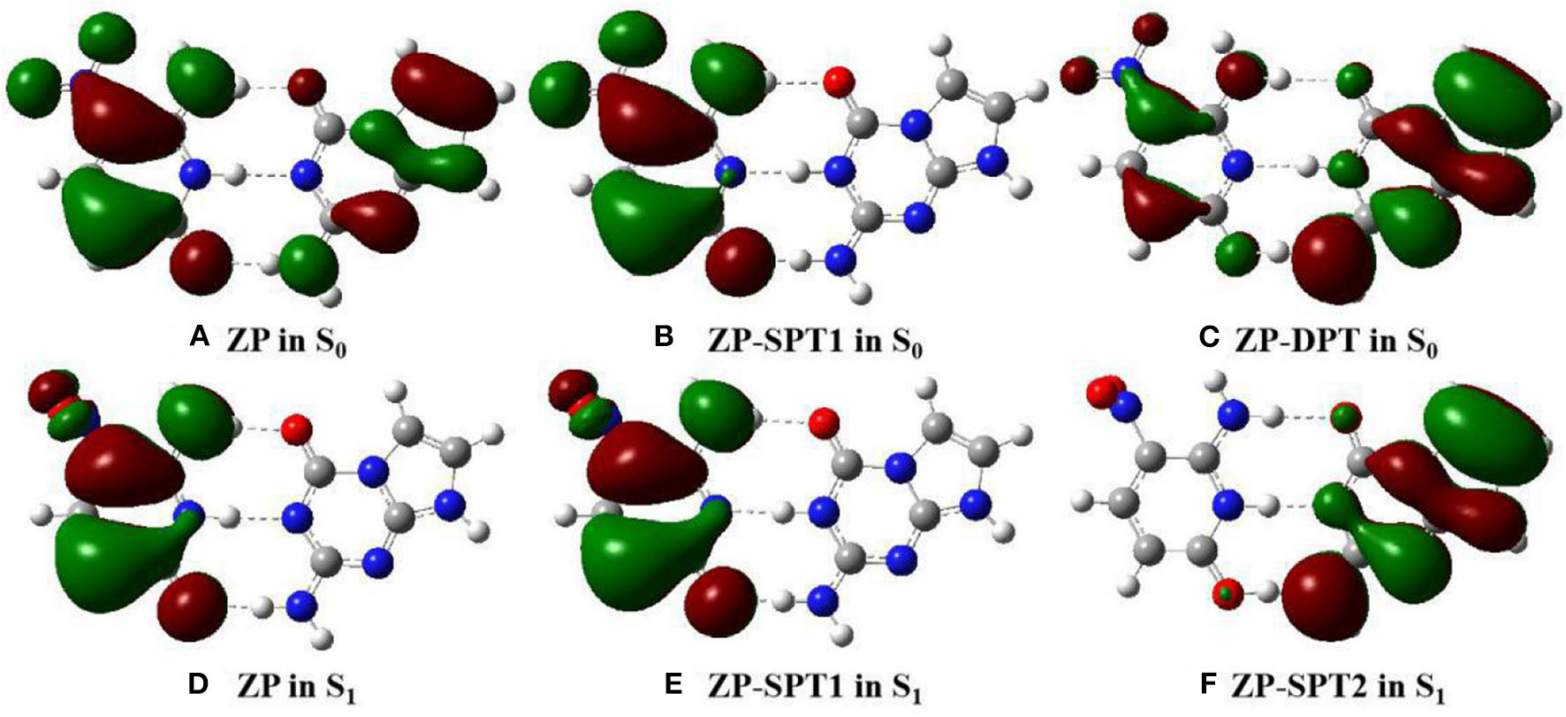
View of HOMO for the normal ZP and tautomers in solution at B3LYP-D3(BJ)/6-311++G(d,p)/IEFPCM level. The green opaque shade and red opaque shade are for positive and negative parts of the wave function (isovalue = 0.02), respectively. **(A)** ZP in S_0_. **(B)** ZP-SPT1 in S_0_. **(C)** ZP-DPT in S_0_. **(D)** ZP in S_1_. **(E)** ZP-SPT1 in S_1_. **(F)** ZP-SPT2 in S_1_.

To further explain the mechanism of excited-state proton transfer in the solvent, we calculated the hydrogen bond parameters of the ZP system. The corresponding electronic distribution of ZP and its tautomers are shown in Figure 3. In addition, the parameters of bond length and bond angle related to the intermolecular hydrogen bond are listed in Supplementary Table 2. Herein, we just investigated the hydrogen bond N1-H1···N2, because it is the primary reaction path in both S_0_ and S_1_ states. Upon photo-excitation, the bond length of N1-H1 is changed from 1.04 Å (S_0_) to 1.05 Å (S_1_), while the bond length of the hydrogen bond H1···N2 is changed from 1.86 Å (S_0_) to 1.78 Å (S_1_). Meanwhile, the bond angle of N1-H1···N2 is changed from 179.0° (S_0_) to 179.2° (S_1_). These phenomena indicate that the intermolecular hydrogen bond N1-H1···N2 is enhanced in the S_1_ state, which provides the driving force for H1 proton transfer in the excited state.

To further reveal the intermolecular hydrogen bonding interactions of ZP in real space, the RDG versus sign(λ_2_)ρ is displayed via the Multiwfn and the VMD programs (see Supplementary Figure 5). The relevant formulas can be written as

(1) $$\begin{array}{l} \text{RDG}\left(r\right)=\frac{1}{2{\left(3\pi^{2}\right)}^{{\;}^{1}{/}_{3}}}\frac{\left|\nabla \rho \left(r\right)\right|}{\rho {\left(r\right)}^{{\;}^{4}{/}_{3}}}, \end{array}$$

(2) $$\begin{array}{l} \Omega \left(r\right)=\text{sign}\left(\lambda_{2}\left(r\right)\right)\rho \left(r\right) \end{array}$$

in which λ_2_(r) reflects the type of interaction and the electron density ρ(r) is used to give the strength of interaction. Besides, the sign(λ_2_)ρ is a good indicator to characterize the interaction strength (Johnson et al., 2010). Specifically, if the value of sign(λ_2_)ρ is negative, the interaction is attractive and is zero meaning that there is a weak interaction, while a positive value of sign(λ_2_)ρ is an indication of non-bonding repulsive interaction. The spike peak of ZP is located around −0.03 a.u. to −0.04 a.u. in the S_0_ state, while the S_1_ state spike peak is between −0.04 a.u. and −0.05 a.u., and the isosurface of the hydrogen bond H1···N2 in S_1_ state is deeper blue distinctly than that in S_0_ state. These calculations indicate that the H1···N2 is strengthened upon photo-excitation. Similar trends are considered in the topology structure (Supplementary Figure 6 and Supplementary Table 5) and the IR spectra (Supplementary Figure 7) of ZP. We also calculated the ESP along the N1-H1···N2 proton transfer path (Supplementary Figure 8) to recognize the electrostatic interaction between molecules. The related results show the ESP of the H1 proton in S_1_ is significantly higher than that in S_0_, which leads to an easier proton transfer from N1 to N2 in S_1_ state. However, the ESP of ZP-SPT1 in the S_0_ state is much higher than that in S_1_ state, indicating the reverse proton transfer is more likely to take place in S_0_ state. Similar trends can also be observed in the differences of atom charges in the S_0_ and S_1_ states. As shown in Supplementary Table 6, the charge of N1 is changed form −0.268 (S_0_) to −0.265(S_1_), while the charge of N2 is changed form −0.424 (S_0_) to −0.438 (S_1_), which has contributions to the H1 proton transfer in the excited state.

In summary, the solvent effect can decrease the potential barriers of single proton transfer, and thus promote the reaction in both S_0_ and S_1_ states. On the other hand, we presented the different trends of proton transfer between ZP and natural base pair GC, which has similar three intermolecular hydrogen bonds like ZP. Although the most easily transferred site is also the H1 proton, the photophysical properties of ZP are different from the GC. Previous studies have proposed a coupled intermolecular electron-proton transfer mechanism for ultrafast electronic deactivation of the GC base pair (Supplementary Figure 9A calculated at CASSCF/CASSPT2 level), which demonstrates the barrierless minimum energy pathway in S_1_ state finally leads to a conical intersection with the S_0_ state at the N1-H1 bond length of about 2.35 Å, and the S_1_ state population returns to S_0_ and the initial Watson-Crick structure is restored. However, the PESs of the S_0_ and S_1_ states of ZP are almost parallel (Supplementary Figures 9B–11), which supports ZP having a longer lifetime in the S_1_ state and possessing weaker photostability than that of natural base pair GC.

### Rotation Modulation of the Electronic Transition

The optimized geometries and related electron distributions for the normal ZP and tautomers are depicted in Figure 3. It can be seen that the canonical ZP Watson-Crick configuration in the S_0_ state is a co-planar structure, in which electrons are delocalized on two bases. However, the rotation of the NO_2_ group on the C5 position of the Z base makes the system non-planar in the S_1_ state, and the electrons concentrated on the Z-base. Obviously, the nitro rotation dominates the transient changes of the electron distributions. It is well-known that as one of the molecular inherent properties, the structural vibrations are persistent, which has been proved to be an important role in the geometries as well as the electronic properties. Accordingly, it is essential to monitor the change of electronic properties along with the geometrical distortions. Here, we considered the configuration of nitro rotation mode, which is quantified by dihedral angle δ(O6-N7-C8-C9).

**Figure 4:**
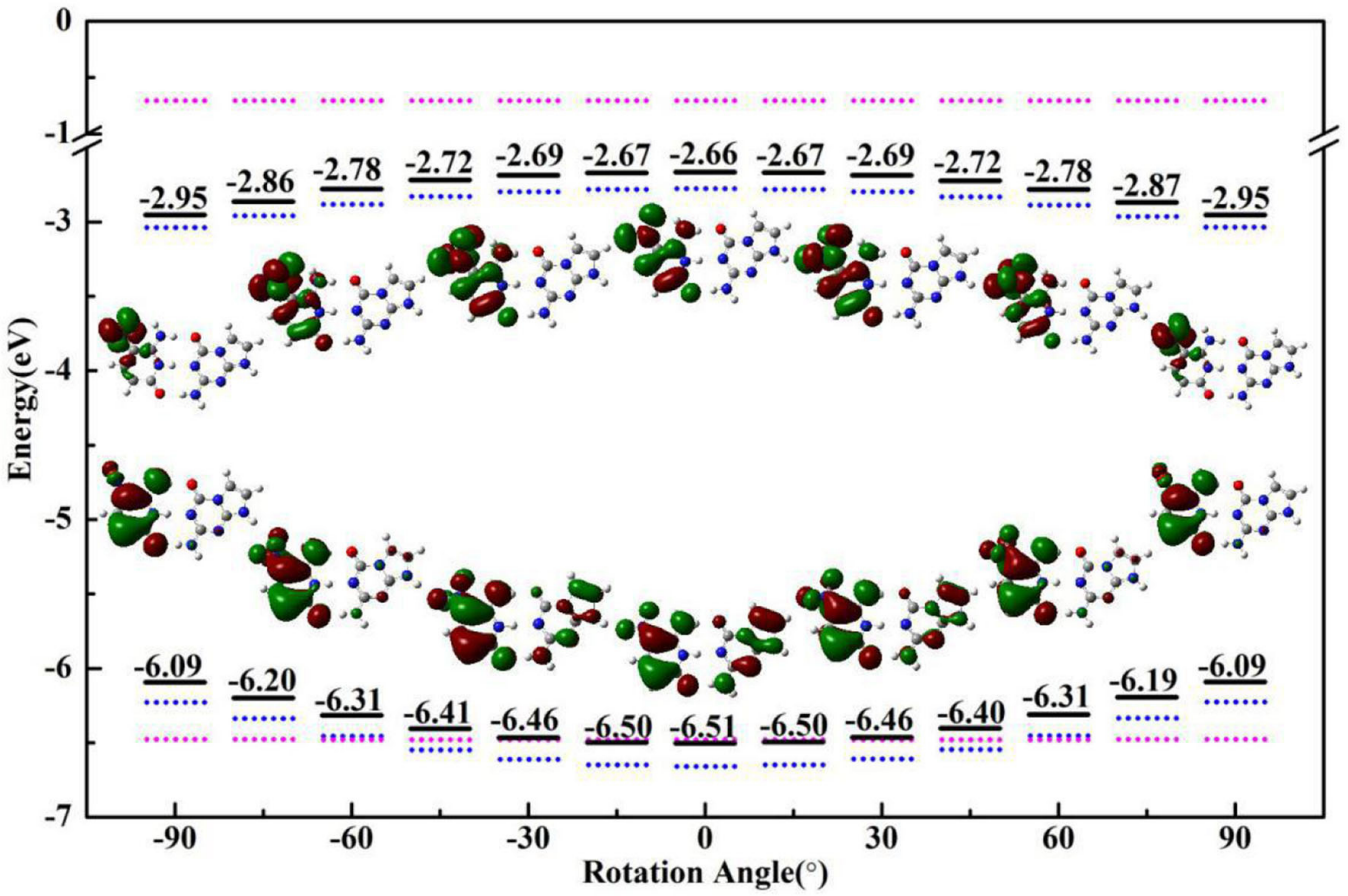
The HOMO/LUMO energy and electron distribution of ZP (black solid line), single Z (blue dotted line) and P (pink dotted line) with the rotational displacements of −90°, −60°, −30°, 0°, 30°, 60°, 90°.

**Figure 5:**
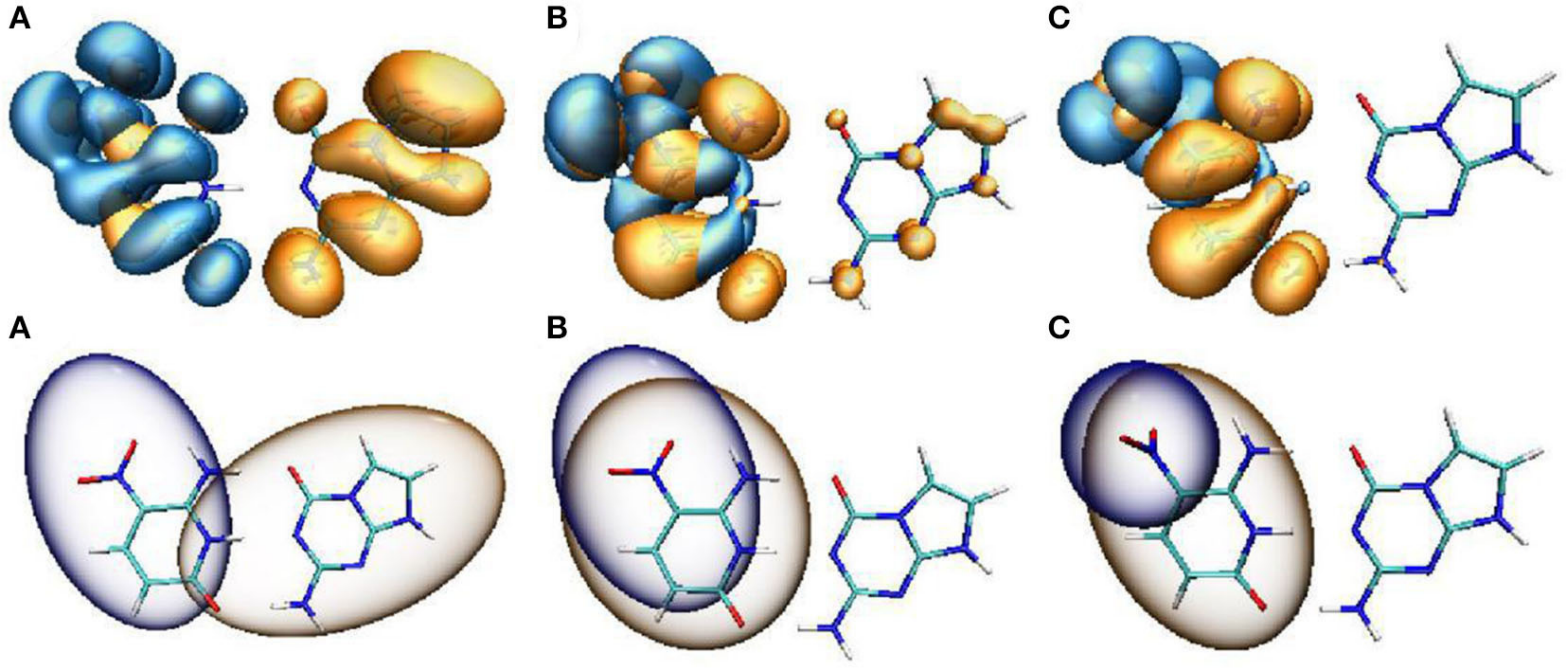
Hole-electron distribution (upper panel) and the C_hole_-C_ele_ diagrams (lower panel) of ZP with the rotational displacements of 0° **(A)**, 45° **(B)**, 90° **(C)**. Orange and blue regions (isovalue = 0.0002) denote the electron distributions and the holes, respectively.

As a spectral indicator of electronic properties of ZP, the variation tendency of the absorption maximum with the nitro rotation is recorded (Supplementary Figure 12). The calculated results shows a continuous red-shift with the enlargement of the dihedral angles δ(O6-N7-C8-C9) and thus indicates a broad absorption spectrum (Supplementary Figure 13). To examine the electron excitation characteristics, we plot the HOMO and LUMO of the ZP base pair. As seen from Figure 4, the electron in both the HOMO and LUMO transfers from the aromatic ring to the nitro group with the enlargement of the dihedral angles. Besides, the hole-electron analysis is also utilized to reveal the nature of electron excitation (i.e., the departure and arrival of the excited electrons). As shown in Figure 5, the ZP undergoes the charge transfer excitation at the most stable geometry in S_0_ with δ = 0°, where the electrons are transferred from P base to Z base. However, the vertical electronic excitation induced charge transfer characteristics become increasingly weak with the enlargement of the dihedral angles. It can be seen that both electrons and holes convergence to the nitro group accompanied with the increase of the rotational displacements, and thus lead to an easier electronic transition from S_0_ to S_1_.

An analysis of HOMO-LUMO gap is also particularly associated with the absorption characters. In general, a small energy gap is advantageous to the electronic transition from HOMO to LUMO. As shown in Figure 4, the HOMO-LUMO gap calculated at the optimized geometries with different dihedral angles in S_0_ state gradually decreases with the increase of the nitro rotation, which is consistent with the red-shift in the absorption spectra. Specifically, the HOMO energies increase with the augment of the dihedral angles, while the LUMO energies decrease with the increase of the dihedral angles, which leads to the narrowing of the HOMO-LUMO energy gap (consistent with the energy difference between the S_0_ and vertical S_1_ states as shown in Supplementary Figure 14) and thus gives rise to the significant red-shift in the absorption spectra. Furthermore, the variation trends of the orbital energies of the separated Z-base and P-base were presented. The structures of single Z-base and P-base are extracted from the optimized geometries of ZP corresponding to different dihedral angles. It can be seen that the HOMO and LUMO energies of Z-base gradually increase and decrease along the positive and negative displacement directions, respectively. However, the orbital energy of P-base is insensitive to the nitro rotation, which remains relatively a constant. These observations indicate the variation of the orbital energies of Z-base induced by the nitro rotation is responsible for the spectral red-shift of ZP base pair. In fact, it is understandable because the orbital energy is an effective parameter to measure the electron-binding ability of a molecule. When the orbital energy is low, the ability of this molecule in binding electron is relatively large, which indicates an electron is prone to be captured. Obviously, the nitro rotation enhances binding ability of LUMO and recedes electron-binding ability of HOMO, respectively, which is conducive to the vertical electronic excitation. In a word, ZP possesses charge-transfer character due to the rotation-induced electron localization on the nitro group, and the transition of ZP from S_0_ to S_1_ is also regulated by the molecular structural fluctuations accompanied with the nitro rotation. The above conclusions have been examined at the wB97-XD and M06-2X functionals (Supplementary Figures 15, 16).

## Conclusion

In this paper, we investigated the intermolecular proton-transfer mechanism and photoisomerization of artificial base pair-ZP. Our results demonstrate that the double proton transfer takes place in a concerted way both in the S_0_ and S_1_ states in the gas phase. Under the water environment, the stepwise mechanism is more favorable along the S_0_-PES and only single proton transfer is available in the S_1_ state. The solvent effect promotes the single proton transfer in both S_0_ and S_1_ states by decreasing the activation energy and stabilizing the products. In addition, the proton transfer reaction is more likely to occur in the S_1_ state because of the excited-state hydrogen bond enhancement. Unlike the excited-state ultrafast deactivation process of the natural bases, there is no conical intersection along the proton transfer coordinate of ZP, which suggests ZP having a longer lifetime in the S_1_ state and possessing weaker photostability than that of natural base pair GC. Corresponding electron distribution reveals that the lowest excited singlet state of ZP possesses charge transfer characters, where the electron transfers from the aromatic ring to the nitro group induced by the photoexcitation. Moreover, we characterized the molecular vibration effect on the electronic excitation. It can be seen that the absorption maximum shows a continuous red-shift with the increase of the dihedral angles δ(O6-N7-C8-C9) due to the narrowing HOMO-LUMO energy gap with the nitro rotation, which indicates a broadened absorption spectrum in contrast to the case of natural base pairs. It can be concluded that the electronic transition of ZP from S_0_ to S_1_ state can be tuned by the rotation-induced structural distortion accompanied with the electron localization on nitro group. This work provides an in-depth understanding on the biological process involved with artificial bases, which may also trigger more promising application prospects on the design of biological drug based on unnatural bases base pairs.

## Data Availability Statement

The raw data supporting the conclusions of this article will be made available by the authors, without undue reservation.

## Author Contributions

XC, YZ, and ZL carried out the *ab initio* calculation. XC analyzed the results and wrote the manuscript. QM and CZ supervised this project. All authors contributed to the article and approved the submitted version.

## Conflict of Interest

The authors declare that the research was conducted in the absence of any commercial or financial relationships that could be construed as a potential conflict of interest.
