# Supplementary material for: Proton Transfer and Nitro Rotation Tuned Photoisomerization of Artificial Base Pair-ZP
Source: Front Chem. 2020 Nov 30;8:605117. doi: 10.3389/fchem.2020.605117 (PMC7734142; doi:10.3389/fchem.2020.605117)
Supplement: Supplementary file 1 [file Data_Sheet_1.pdf]

Supplementary Material

Contents

1 Optimized structures, energies and some important parameters involved in three intermolecular hydrogen bonds in gas phase and solution: Figures S1-S3 and Tables S1-S3.

2 Potential energy curves with the explicit solvent molecules (5H_2O molecules): Figure S4 and Table S4.

3 RDG, Topological analysis, IR, electrostatic potential and Mulliken charge of ZP: Figures S5-S8 and Tables S5-S6.

4 Potential energy curves calculated with the wB97-XD and M06-2X functionals and wave function methods: Figures S9-S11.

5 Structures, energies and absorption maxima of ZP: Figures S12-S16.

6 References

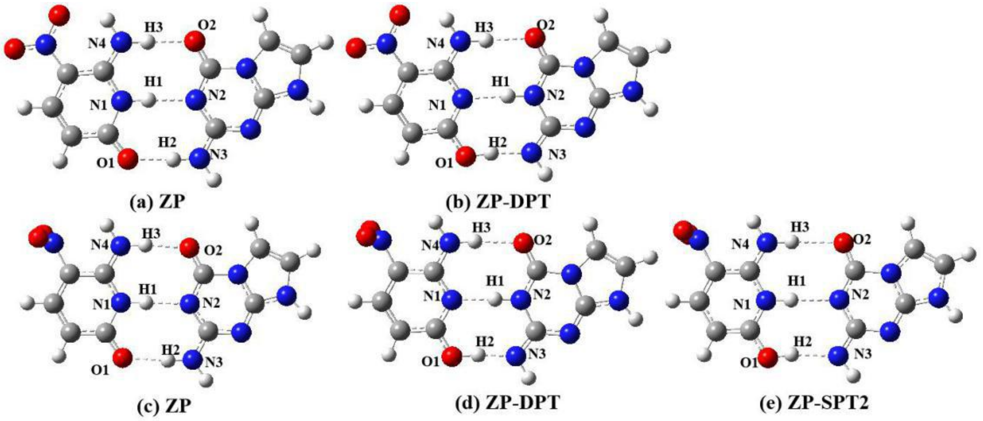

FIGURE S1 Optimized constructions of ZP in S_0 ((a) and (b))and S_1 ((c), (d) and (e)) states in the gas phase.

TABLE S1 Primary bond lengths (Å) and bond angles (°) of ZP, ZP-SPT2, ZP-DPT in S_0 and S_1 states, respectively

	**ZP**		**ZP-DPT**		**ZP-SPT2**
	**S_0**	**S_1**	**S_0**	**S_1**	**S_1**
**N1-H1(Å)**	1.037	1.044	1.813	1.737	1.023
**H1-N2(Å)**	1.868	1.800	1.048	1.049	1.975
**O1-H2(Å)**	1.780	1.870	1.024	1.062	0.977
**H2-N3(Å)**	1.029	1.022	1.618	1.520	1.898
**N4-H3(Å)**	1.024	1.028	1.015	1.020	1.008
**H3-O2(Å)**	1.851	1.810	1.955	1.964	2.114
**N1-H1-N2(°)**	177.644	179.359	177.585	177.260	179.593
**O1-H2-N3(°)**	176.644	174.763	172.510	174.261	170.254
**N4-H3-O2(°)**	179.719	174.115	178.446	174.528	176.112

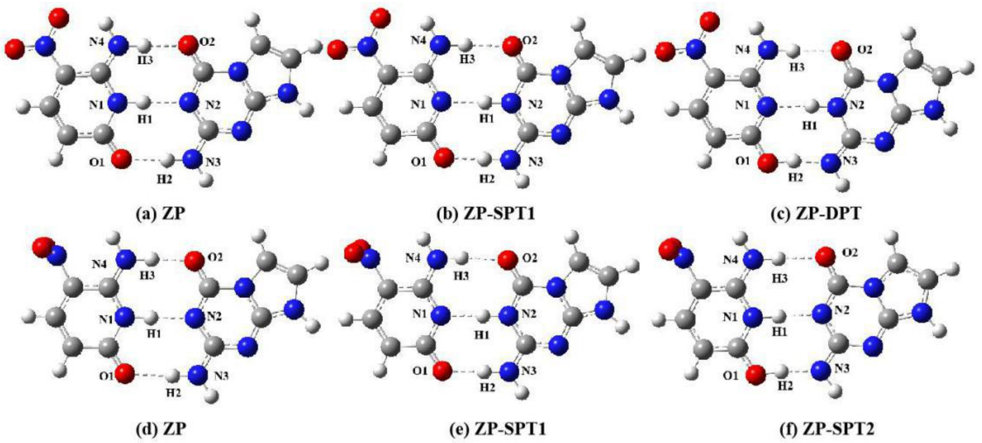

FIGURE S2 Optimized constructions of ZP in S_0 ((a), (b) and (c))and S_1 ((d), (e) and (f)) states in the solution.

TABLE S2 Primary bond lengths (Å) and bond angles (°) of ZP, ZP-SPT1, ZP-SPT2, ZP-DPT in S_0 and S_1 states, respectively

	**ZP**		**ZP-DPT**		**ZP-DPT**	**ZP-SPT2**
	**S_0**	**S_1**	**S_0**	**S_1**	**S_0**	**S_1**
**N1-H1(Å)**	1.042	1.052	1.761	1.740	1.817	1.049
**H1-N2(Å)**	1.859	1.777	1.066	1.063	1.042	1.754
**O1-H2(Å)**	1.847	1.922	1.631	1.724	1.066	1.030
**H2-N3(Å)**	1.022	1.017	1.048	1.035	1.503	1.593
**N4-H3(Å)**	1.029	1.038	1.015	1.019	1.017	1.019
**H3-O2(Å)**	1.791	1.723	1.915	1.904	1.963	1.911
**N1-H1-N2(°)**	178.985	179.220	179.010	178.660	176.468	178.886
**O1-H2-N3(°)**	176.887	175.129	179.508	178.518	172.201	173.926
**N4-H3-O2(°)**	177.807	177.310	178.521	173.477	177.942	179.636

TABLE S3 The calculated energies of all geometric configurations in the gas phase and solution.

		**Energy(a.u.)**			
	**State**	**ZP**	**ZP-SPT1**	**ZP-SPT2**	**ZP-DPT**
**Gas Phase**	**S_0**	-1126.389232	/	/	-1126.369814
	**S_1**	-1126.303148	/	-1126.280744	-1126.282825
**Aqueous Phase**	**S_0**	-1126.414877	-1126.403575	/	-1126.394694
	**S_1**	-1126.335681	-1126.331358	-1126.295805	/

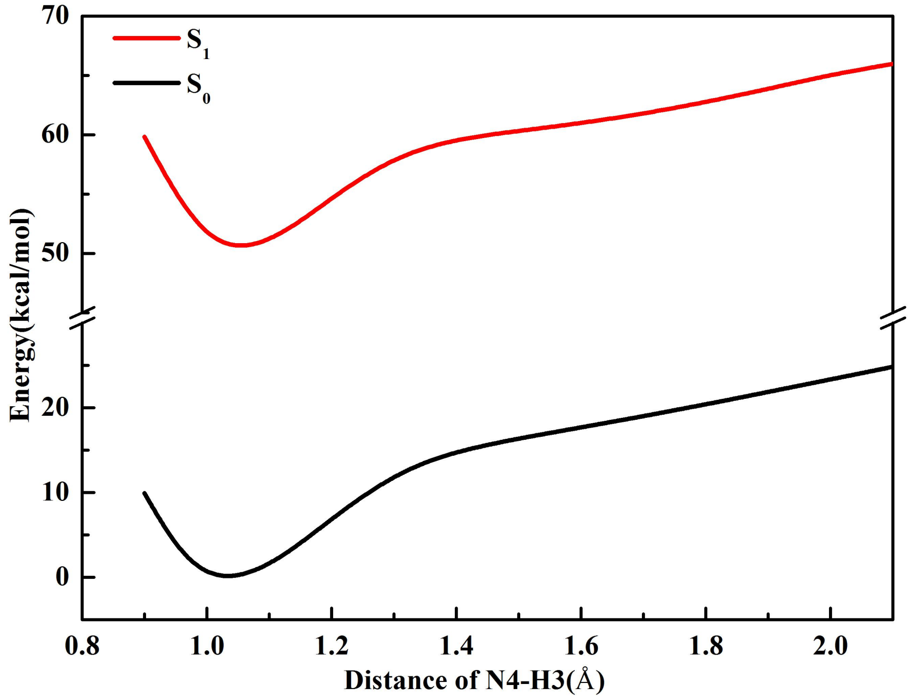

FIGURE S3 Potential energy curves in the S_0 and S_1 states for ZP as a function of the N4-H3 bond length.

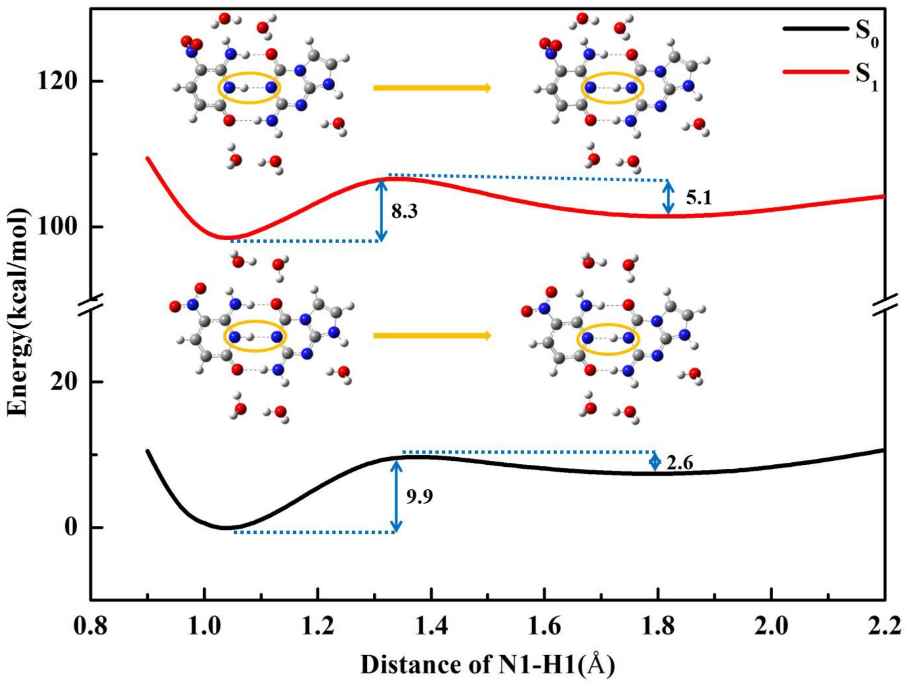

FIGURE S4 The relaxed potential energy curves in the S_0 and S_1 states for ZP as a function of the N1-H1 bond length in solution (with the first hydration shell ($5H_2O$ molecules) and the IEFPCM model) at B3LYP-D3(BJ)/6-311++G(d,p)/IEFPCM level.

TABLE S4 The potential energy barrier of proton H1 transfer process (ΔE) and reverse reaction (ΔE_{re}) in the gas phase and solution (IEFPCM/IEFPCM+5H_2O) at B3LYP-D3(BJ)/6-311++G(d,p)/IEFPCM level.

	**Structure**	**ΔE(kcal/mol)**	**ΔE_{re}(kcal/mol)**
**Gas Phase**	WC-ZP-S_0	/	/
	WC-ZP-S_1	/	/
**IEFPCM**	WC-ZP-S_0	8.9	1.7
	WC-ZP-S_1	5.9	3.2
**IEFPCM+5H_2O**	WC-ZP-S_0	9.9	2.6
	WC-ZP-S_1	8.3	5.1

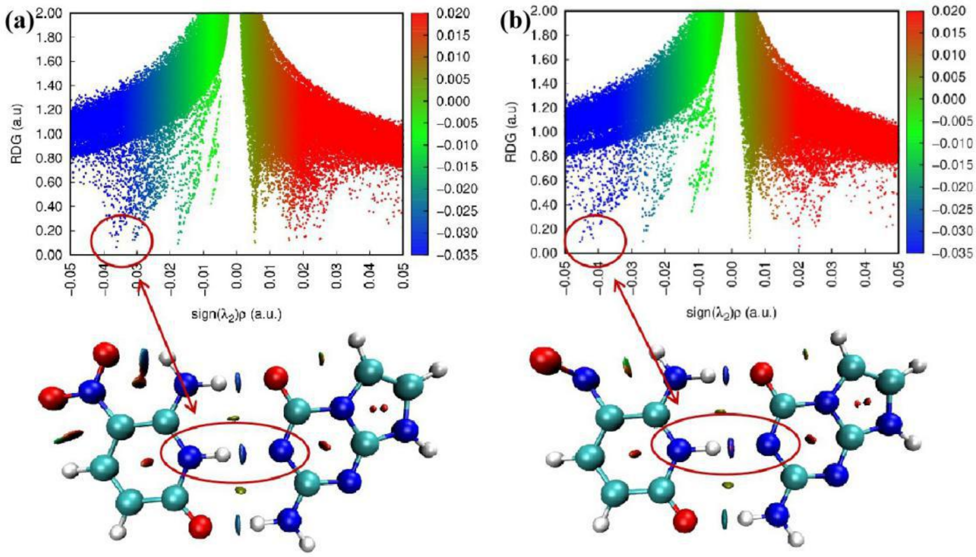

FIGURE S5 The scatter plot and gradient isosurface of ZP in S_0 and S_1 states at B3LYP-D3(BJ)/ 6-311++G(d,p)/ IEFPCM level. (a) S_0 state; (b) S_1 state.

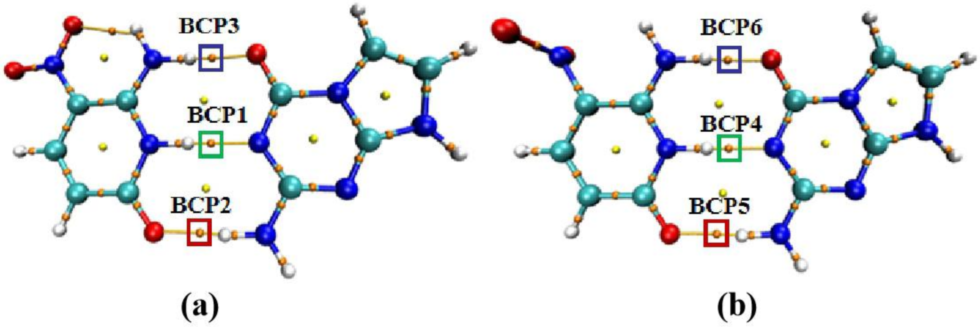

FIGURE S6 Topological analysis diagrams of ZP including bond paths and bond critical points in S_0 and S_1 states (a) S_0 state ; (b) S_1 state.

TABLE S5 Obtained parameters of bond critical points (BCPs) related to hydrogen bonds in the ZP systems

	ZP-S_0			ZP-S_1		
	BCP1(HB1)	BCP2(HB2)	BCP3(HB3)	BCP4(HB1)	BCP5(HB2)	BCP6(HB3)
$\rho(r)^a$ (a.u.)	0.0378	0.0312	0.0363	0.0456	0.0267	0.0424
$V(r)^b$ (a.u.)	-0.0289	-0.0251	-0.0306	-0.0384	-0.0197	-0.0386
$E_{HB}{}^c$ (KJ/mol)	-32.175	-26.015	-30.775	-39.456	-21.815	-36.469
$\nabla^2\rho(r)^d$ (a.u.)	0.0922	0.1094	0.1185	0.0997	0.0924	0.1336
$G(r)^e$ (a.u.)	0.0260	0.0262	0.0301	0.0317	0.0214	0.0360
$H(r)^f$ (a.u.)	-0.0029	0.0012	-0.0005	-0.0068	0.0017	-0.0026

[a] $\rho(r)$ is electron density.

[b] $V(r)$ is the potential energy density.

[c] E_{HB} is hydrogen bond energy.

[d] $\nabla^2\rho(r)$ is Laplacian of electron density.

[e] $G(r)$ is kinetic energy density.

[f] $H(r)$ is the total electron energy density.

In topology analysis language, the points at which the gradient norm of function value is zero (except at infinity) are called as critical points (CPs). According to Bader's theory (Bader and Essén, 1984), the existence of bond paths and the identification of CPs in equilibrium geometry are sufficient conditions for obtaining the interaction between two main atoms. BCP1, BCP2, and BCP3 represent the bond critical points of ZP (HB1), ZP (HB2), and ZP (HB3) in the S_0 state, respectively. In the meanwhile, BCP4, BCP5, and BCP6 represent the bond critical points of ZP (HB1), ZP (HB2) and ZP (HB3) in the S_1 state, respectively. The electron density ρ (r) and the potential energy density V (r) at BCP position are closely related to the strength of chemical bond. In general, for the same kind of the chemical bond, the larger the ρ (r) and the more negative the V (r) at the BCPs, the stronger the interaction between the two atoms connected by the bond path. The value of ρ (r) changes from 0.0378 a.u. to 0.0456 a.u. with BCP from BCP1 (S_0) to BCP4 (S_1). At the same time, the value of V (r) changes from -0.0289 a.u. to -0.0384 a.u. These results indicate that intermolecular hydrogen bond H1···N2 is enhanced from the S_0 to S_1 state. They can also be explained by the increase in the bond energy of hydrogen bonds (E_{HB}), which is obtained by the following formula

$$E_{HB} = -223.08\rho(r) + 0.7423. \quad (1)$$

Eq. (1) was put forward by Lu et al. in 2019 (Emamian et al., 2019). It can be used to calculate accurately the strength of the hydrogen bond. In a word, the topological analysis further proves that the intermolecular hydrogen bond N1-H1···N2 is enhanced from the S_0 to S_1, which promotes the transfer of proton H1.

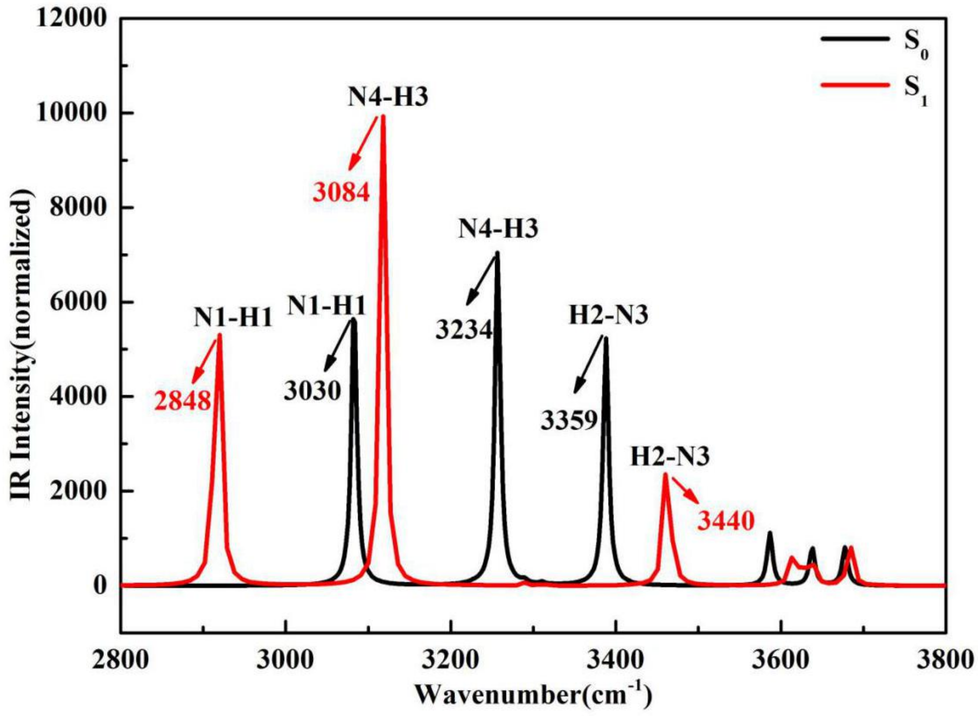

FIGURE S7 Theoretical IR spectra of the normal ZP at the spectral region of N-H stretching bands in S_0 and S_1 states.

The IR spectra of chemical bonds connected with hydrogen bonds is calculated to explore the excited-state hydrogen bond interactions (Zhao and Han, 2007; Zhang et al., 2016). It can be seen that the calculated frequency of the N1-H1 is 3030 cm^{-1} and 2848 cm^{-1} in the S_0 and S_1 states, respectively. The red-shift about 182 cm^{-1} reveals the intermolecular hydrogen bond N1-H1···N2 is reinforced in the S_1 state, which promotes the exciting state single-proton transfer reaction of the ZP system.

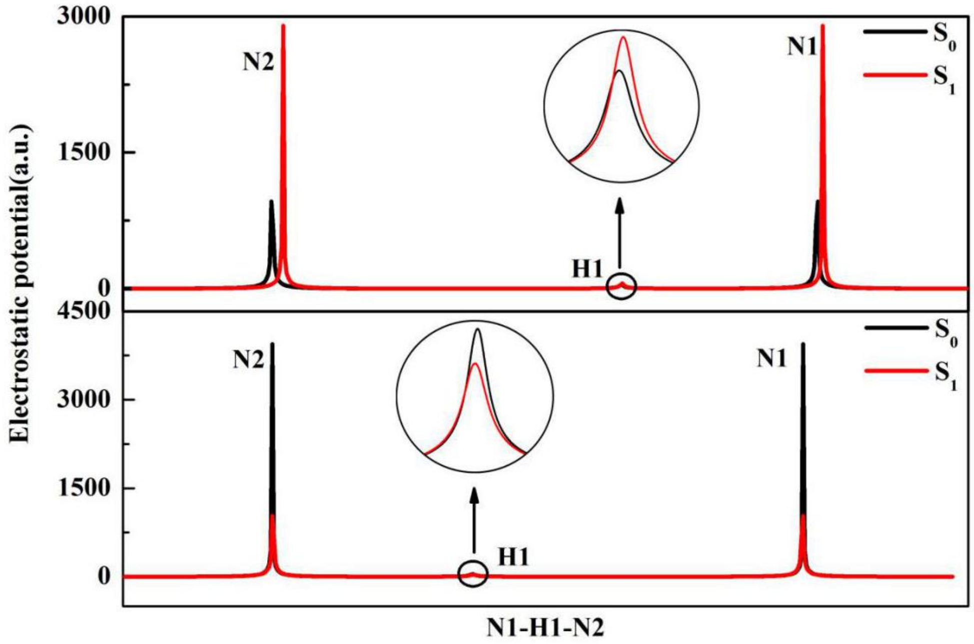

FIGURE S8 The electrostatic potential of ZP (upper) and ZP-SPT1 (lower) in S_0 and S_1 states along the N1-H1-N2 path.

TABLE S6 Mulliken charge (a.u.) of proton donor (N1) and proton acceptor (N2) in the S_0 and S_1 states

State	N1	N2
**S_0**	-0.268	-0.424
**S_1**	-0.265	-0.438

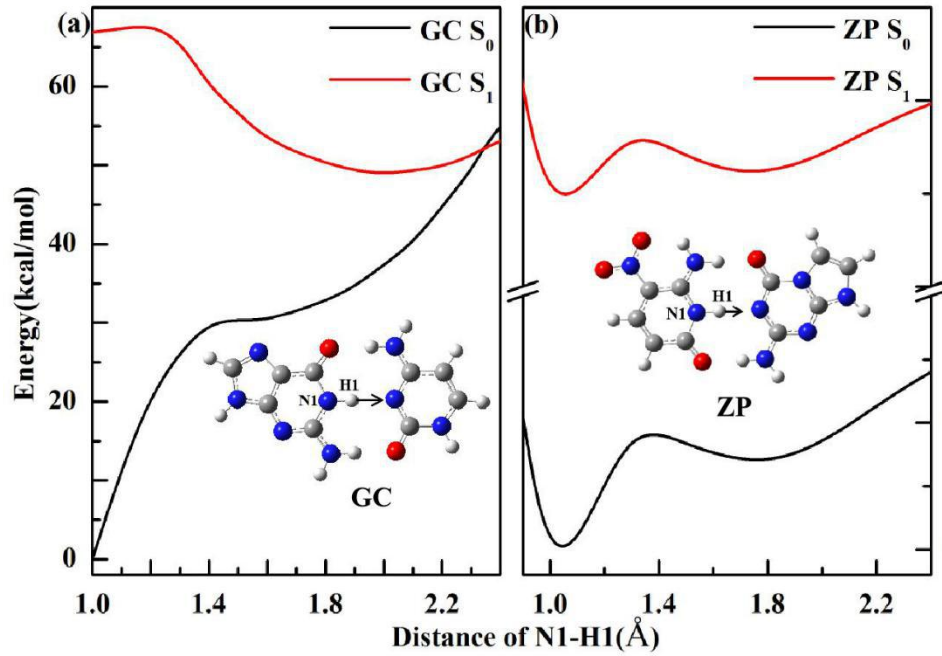

FIGURE S9 Potential energy curves of the S_0 and S_1 states as function of N1-H1 bond lengths in GC (a, calculated at CASSCF/CASSPT2 level) (Sobolewski and Domcke, 2004; Sobolewski et al., 2005) and ZP (b, calculated at B3LYP-D3(BJ) level).

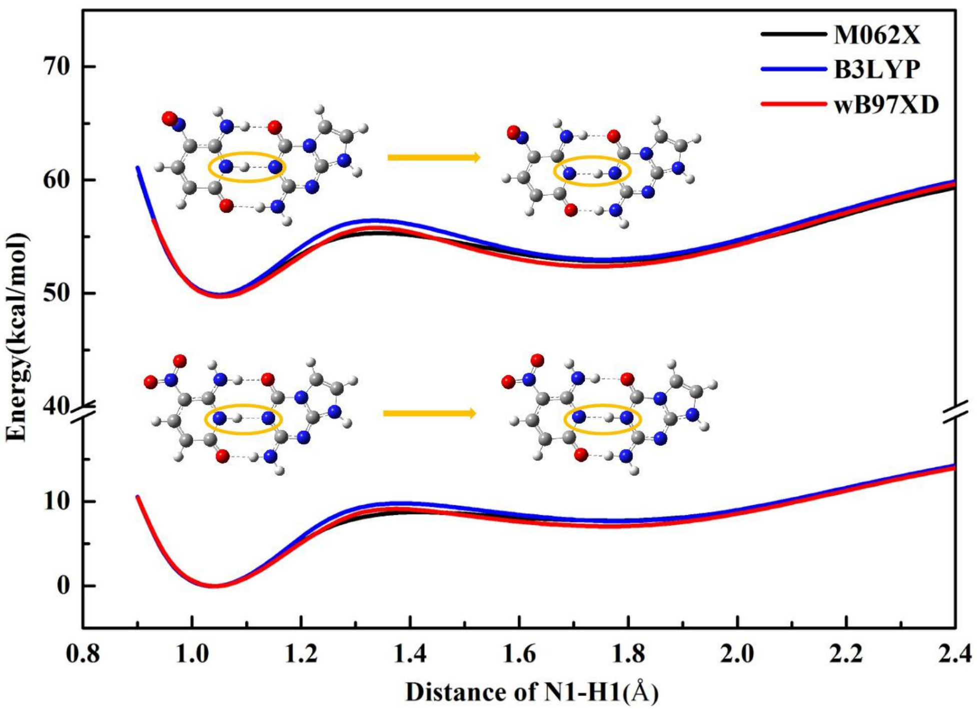

FIGURE S10 Potential energy curves in the S_0 and S_1 states for ZP as a function of the N1-H1 bond length. The upper reflects the potential energy of the S_1 state and the lower corresponds to the S_0 state.

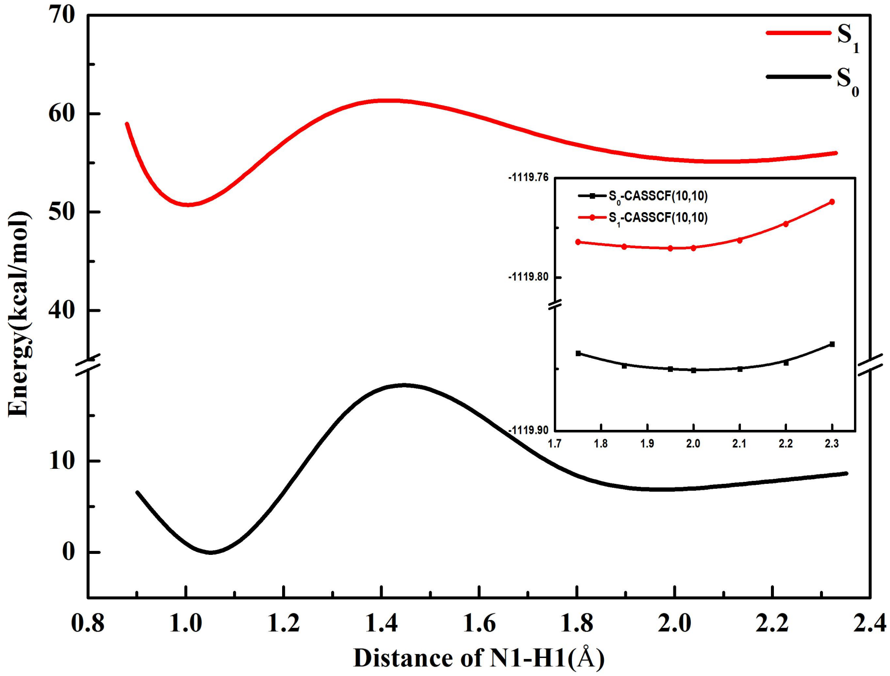

FIGURE S11 Potential energy curves in the S_0 and S_1 states for ZP at the DLPNO-STEOM-CCSD/def2-TZVPP level based on the DFT(S_0)/TDDFT(S_1) optimized geometries. The inset reflects the potential energy around the possible conical intersection region at the CASSCF(10,10)/6-311++G(d,p) level.

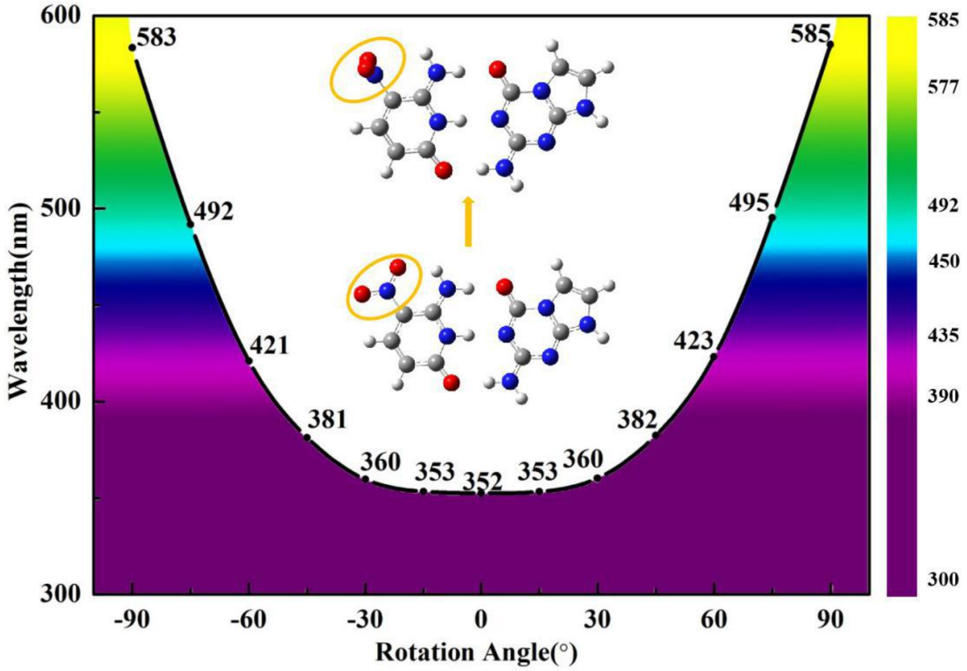

FIGURE S12 The variation trends of absorption maxima with the nitro rotation at B3LYP-D3(BJ)/ 6-311++ G (d, p)/ IEFPCM level.

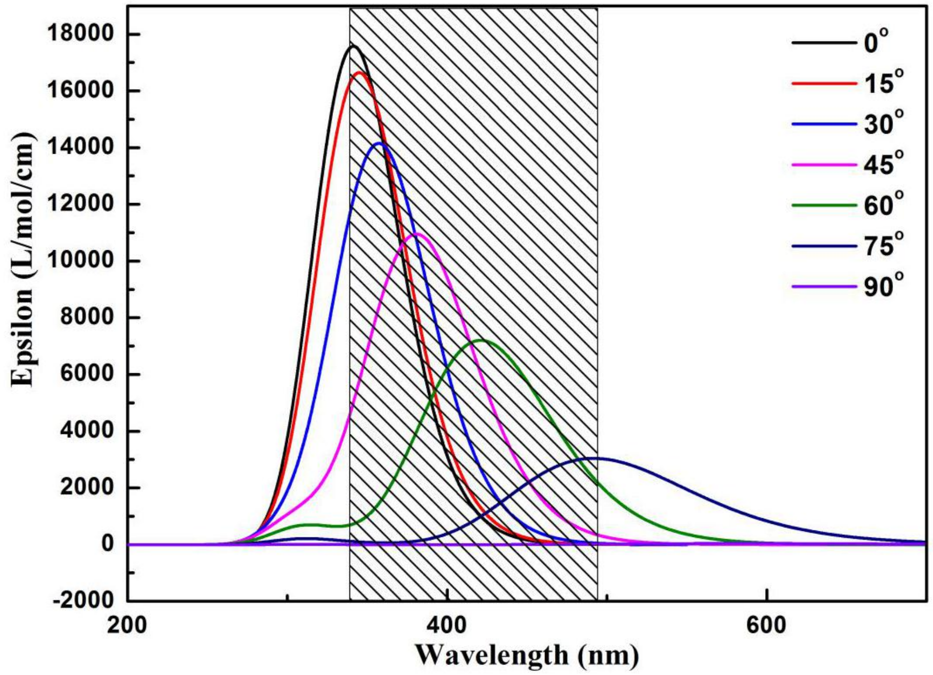

FIGURE S13 The absorption spectra of ZP with the rotational displacements of 0°, 15°, 30°, 45°, 60°, 75°, 90°.

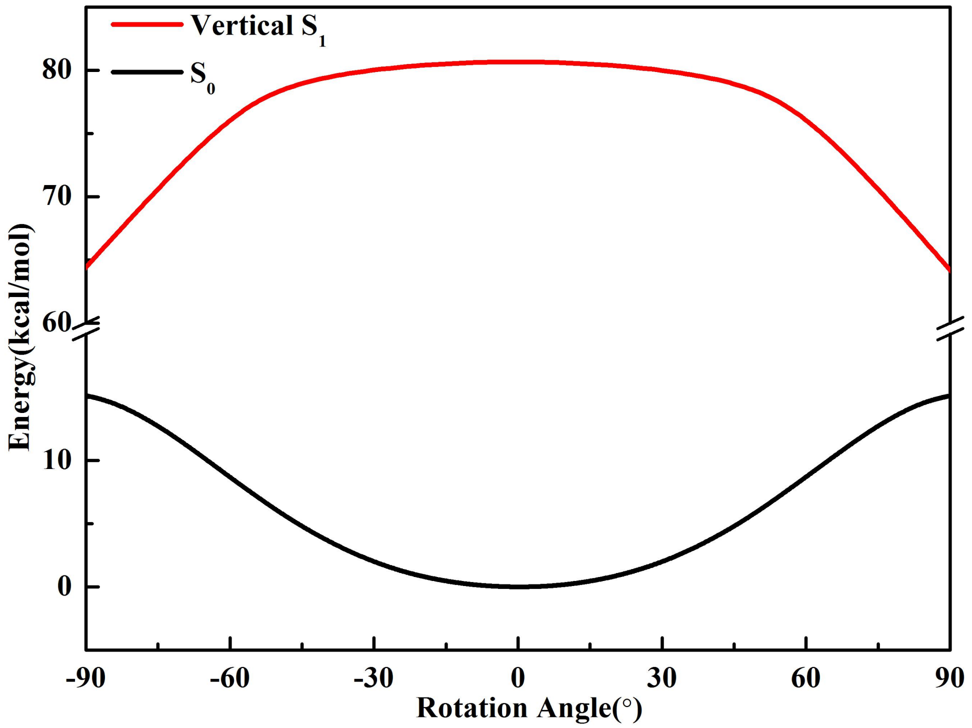

FIGURE S14 The variation trends of S_0 and vertical S_1 energy with the nitro rotation.

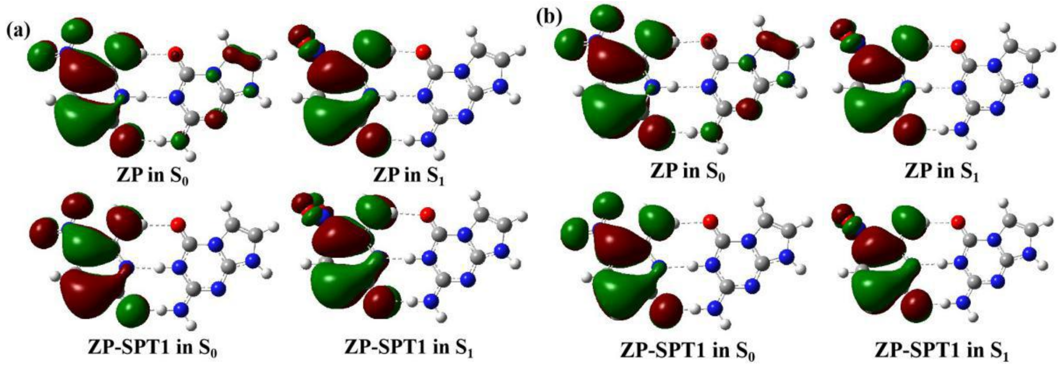

FIGURE S15 The electron distribution of the ZP and ZP-SPT1 in S_0 and S_1 states. The isovalue is set as 0.02. (a) M06-2X/6-311++ G (d, p). (b) wB97-XD/6-311++ G (d, p).

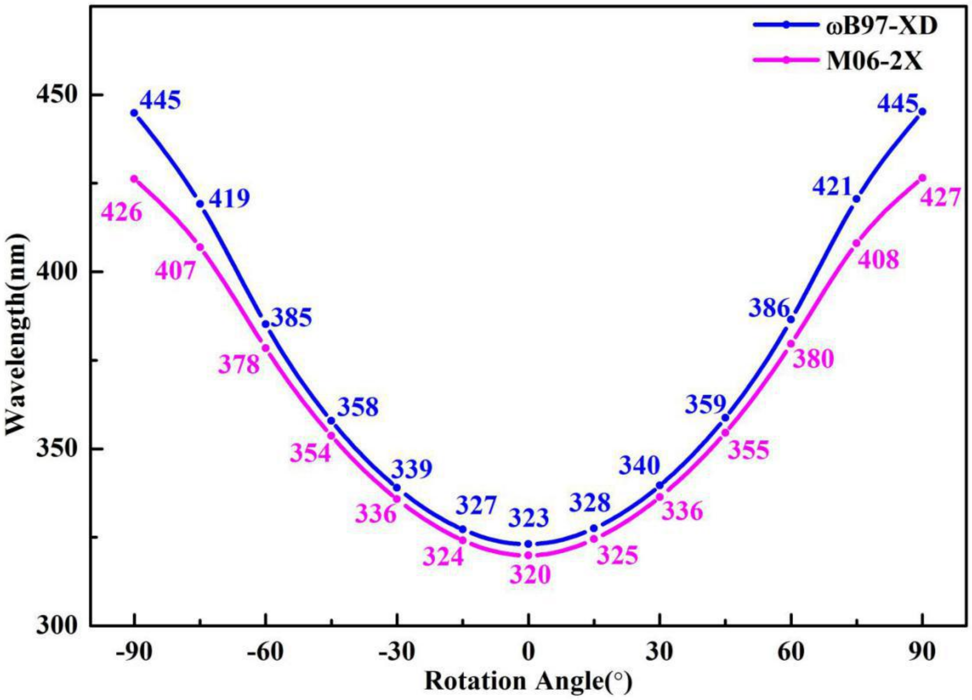

FIGURE S16 The absorption maxima of ZP as the nitro rotation by utilizing the wB97-XD and M06-2X functionals.

References

Bader, R. F. W., and Essén, H. (1984). The characterization of atomic interactions. *J. Chem. Phys.* 80, 1943-1960. doi: 10.1063/1.446956

Emamian, S., Lu, T. , Kruse, H., and Emamian, H. (2019).Exploring Nature and Predicting Strength of Hydrogen Bonds: A Correlation Analysis Between Atoms-in-Molecules Descriptors, Binding Energies, and Energy Components of Symmetry-Adapted Perturbation Theory. *J. Comput. Chem.* 40, 2868-2881. doi: 10.1002/jcc.26068

Sobolewski, A. L., and Domcke, W. (2004). Ab initio studies on the photophysics of the guanine–cytosine base pair. *Phys. Chem. Chem. Phys.* 6, 2763-2771. doi: 10.1039/b314419d

Sobolewski, A. L., Domcke, W., and Hattig, C. (2005). Tautomeric selectivity of the excited-state lifetime of guanine/cytosine base pairs: the role of electron-driven proton-transfer processes. *P. Natl. Acad. Sci. USA* 102, 17903-17906. doi: 10.1073/pnas.0504087102

Zhang, Y. J., Zhao, J. F., and Li, Y. Q. (2016). The investigation of excited state proton transfer mechanism in water-bridged 7-azaindole. *Spectrochim. Acta. A* 153, 147-151. doi: 10.1016/j.saa.2015.08.028

Zhao, G. J., and Han, K. L. (2007). Ultrafast hydrogen bond strengthening of the photoexcited fluorenone in alcohols for facilitating the fluorescence Quenching. *J. Phys. Chem. A* 111, 9218-9223. doi: 10.1021/jp0719659
